# Regulation of ATR-dependent DNA damage response by nitric oxide

**DOI:** 10.1016/j.jbc.2021.100388

**Published:** 2021-02-07

**Authors:** Chay Teng Yeo, Jennifer S. Stancill, Bryndon J. Oleson, Jamie K. Schnuck, Joshua D. Stafford, Aaron Naatz, Polly A. Hansen, John A. Corbett

**Affiliations:** Department of Biochemistry, Medical College of Wisconsin, Milwaukee, Wisconsin, USA

**Keywords:** Nitric oxide, DDR, ATR, beta-cell, metabolism, mitochondria, oxidation, ribonucleotide reductase, ATM, ataxia-telangiectasia mutated protein, ATR, ataxia-telangiectasia and Rad3-related protein, Chk, checkpoint kinases, cPTIO, 2-(4-carboxyphenyl)-4,5-dihydro-4,4,5,5-tetramethyl-1H-imidazolyl-1-oxy-3-oxide, DDR, DNA damage response, DEA/NO, 2-(*N*,*N*-diethylamino)-diazenolate-2-oxide, DMEM, Dulbecco's modified Eagle's medium, DPTA/NO, (Z)-1-[*N*-(3-aminopropyl)-*N*-(3-ammoniopropyl)amino]diazen-1-ium-1,2-diolate, ECAR, extracellular acidification rate, FBS, fetal bovine serum, FCCP, carbonyl cyanide 4-(trifluoromethoxy)phenylhydrazone, H2AX, H2A histone family member X, HRP, horseradish peroxidase, IFN-γ, interferon gamma, IL-1, interleukin-1, iNOS, inducible nitric oxide synthase, KAP1, Krüppel-associated box–associated protein 1, LDH, lactate dehydrogenase, MEF, mouse embryonic fibroblast, NMMA, N^G^-monomethyl-l-arginine, NOS, nitric oxide synthase, OCR, oxygen consumption rate, RNR, ribonucleotide reductase, SSBs, single-strand breaks

## Abstract

We have shown that nitric oxide limits ataxia-telangiectasia mutated signaling by inhibiting mitochondrial oxidative metabolism in a β-cell selective manner. In this study, we examined the actions of nitric oxide on a second DNA damage response transducer kinase, ataxia-telangiectasia and Rad3-related protein (ATR). In β-cells and non–β-cells, nitric oxide activates ATR signaling by inhibiting ribonucleotide reductase; however, when produced at inducible nitric oxide synthase–derived (low micromolar) levels, nitric oxide impairs ATR signaling in a β-cell selective manner. The inhibitory actions of nitric oxide are associated with impaired mitochondrial oxidative metabolism and lack of glycolytic compensation that result in a decrease in β-cell ATP. Like nitric oxide, inhibitors of mitochondrial respiration reduce ATP levels and limit ATR signaling in a β-cell selective manner. When non–β-cells are forced to utilize mitochondrial oxidative metabolism for ATP generation, their response is more like β-cells, as nitric oxide and inhibitors of mitochondrial respiration attenuate ATR signaling. These studies support a dual role for nitric oxide in regulating ATR signaling. Nitric oxide activates ATR in all cell types examined by inhibiting ribonucleotide reductase, and in a β-cell selective manner, inducible nitric oxide synthase–derived levels of nitric oxide limit ATR signaling by attenuating mitochondrial oxidative metabolism and depleting ATP.

The DNA damage response (DDR) represents a series of signaling cascades that are activated when DNA is damaged (1). The three major DDR transducer kinases, ataxia-telangiectasia mutated protein (ATM), ataxia-telangiectasia and Rad3-related protein (ATR), and DNA-dependent protein kinase, belong to the phosphatidylinositol 3-kinase–related kinase family (2). The transducer kinases are activated by the type and extent of DNA damage. ATM and DNA-dependent protein kinase are activated by DNA double-strand breaks, whereas ATR is activated in response to replication stress and DNA single-strand breaks (SSBs) (2). Once activated, they initiate DNA repair, cellular senescence, cell cycle arrest, or proapoptotic pathways (1, 3). Substrates of DDR kinases include the histone variant H2A histone family member X (H2AX) (γH2AX when phosphorylated), checkpoint kinases (Chk) 1 and 2, and Krüppel-associated box–associated protein 1 (KAP1) (4, 5, 6, 7, 8, 9, 10). Chk1 appears to be a selective substrate of ATR, whereas KAP1 is selective for ATM (6, 10).

Recently, we have shown that interleukin-1 (IL-1) stimulates the formation of γH2AX selectively in pancreatic β-cells and not in other cell types found in the islets of Langerhans (11). Its formation is attenuated by nitric oxide synthase (NOS) inhibitors (11, 12), consistent with DNA damage that is induced by nitric oxide in β-cells following inducible nitric oxide synthase (iNOS) expression (13). γH2AX formation in response to nitric oxide is dependent on ATM, as it is not observed in islets isolated from mice deficient in this DDR kinase (11). Consistent with its dual role as a mediator of cellular damage and a protective molecule, when actively produced at iNOS-derived low micromolar levels, nitric oxide inhibits ATM-dependent DDR signaling (14, 15). The inhibition of ATM signaling by nitric oxide is selective for β-cells (14, 15, 16).

The cell type selective inhibition of ATM by nitric oxide is associated with metabolic flexibility. In β-cells, glycolysis is coupled to mitochondrial oxidative metabolism such that 90% of the carbons of glucose are oxidized to CO_2_, and the rates of oxidation increase with increasing concentrations of substrate (17, 18, 19). This results in an increase in the ATP/ADP ratio, closure of ATP-sensitive K^+^ channels, membrane depolarization, and Ca^2+^-dependent insulin secretion (17, 18, 19, 20, 21, 22). Nitric oxide mediates the inhibitory actions of cytokines on insulin secretion (23, 24, 25) by attenuating the Krebs cycle enzyme aconitase and complex IV of the electron transport chain (26, 27). Because of the coupling of aerobic and anaerobic metabolisms, β-cell ATP levels are decreased when mitochondrial respiration is inhibited because of a lack of glycolytic compensation (17, 28, 29, 30, 31). In non–β-cells, nitric oxide does not decrease ATP because they have the metabolic flexibility to increase glycolysis (28). Recently, we have shown that the same metabolic pathways responsible for the control of glucose-stimulated insulin secretion are inhibited by nitric oxide, and this results in an attenuation in ATM signaling and the inhibition of DNA damage–induced β-cell apoptosis (14, 28, 32).

In this study, the role of nitric oxide as a regulator of ATR signaling has been examined. ATR is activated by SSBs and the depletion of deoxyribonucleotides causing replication stress (33, 34). Hydroxyurea activates ATR by inhibiting ribonucleotide reductase (RNR) and thereby preventing the formation of deoxyribonucleotides required for DNA synthesis (35, 36, 37). Even though RNR was one of the first enzymes shown to be inhibited by nitric oxide (38, 39), the effects of nitric oxide on ATR signaling have yet to be evaluated. We show that nitric oxide plays a dual role in regulating ATR signaling. In all cell types examined, nitric oxide activates ATR signaling consistent with the inhibition of RNR. Nitric oxide also inhibits ATR signaling in a β-cell selective manner when present at iNOS-derived or low micromolar levels. The β-cell selective inhibition of ATR signaling is associated with the inhibition of mitochondrial oxidative metabolism and depletion of cellular ATP. These novel findings have identified a second DDR kinase that is sensitive to nitric oxide in a β-cell selective manner.

## Results

### Nitric oxide as an activator of ATR-dependent DDR signaling

**Figure 1:**
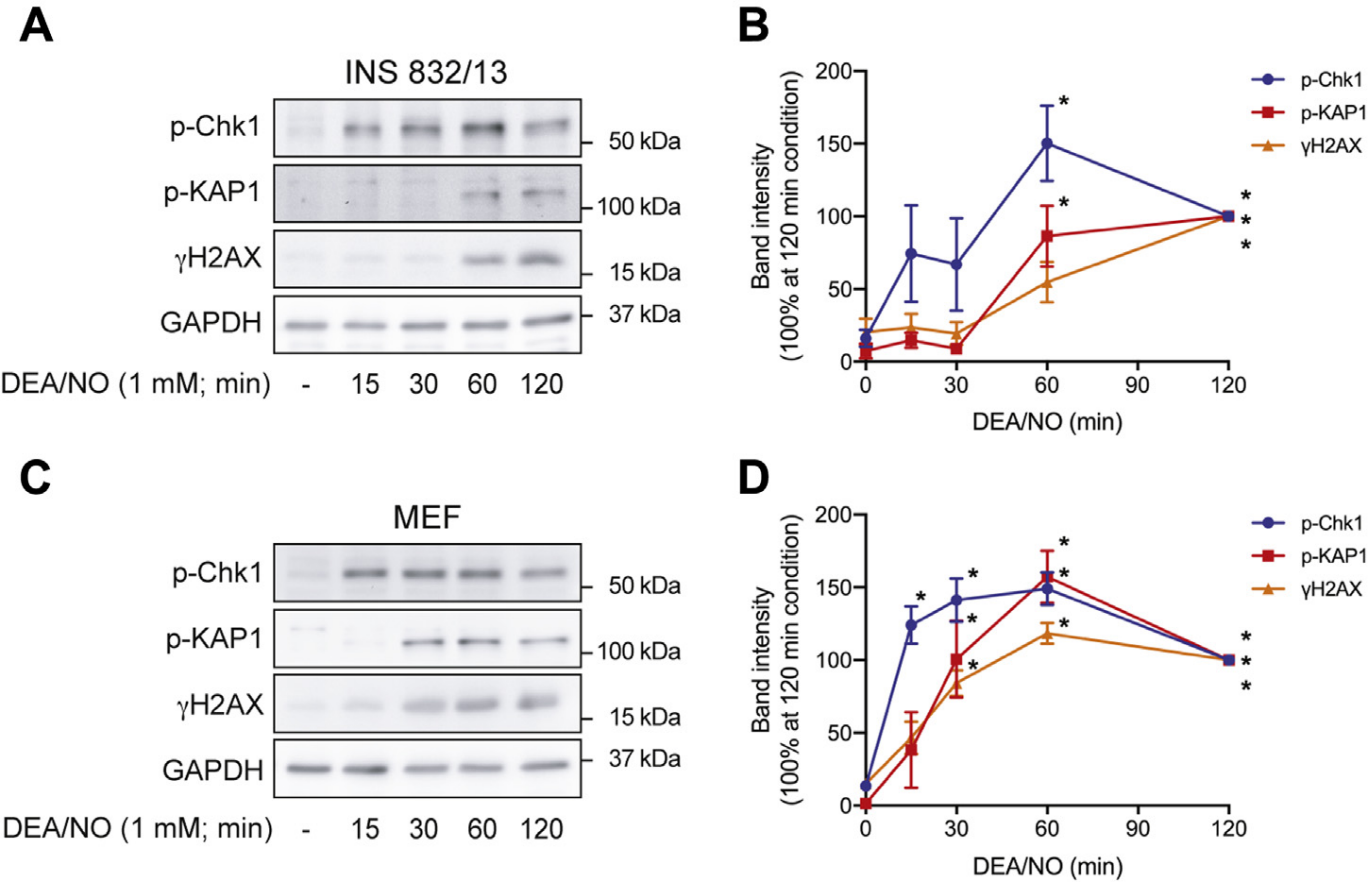
**Nitric oxide activates ATM- and ATR-dependent DDR signaling.** INS 832/13 cells (*A* and *B*) and MEF (*C* and *D*) were treated with DEA/NO for the indicated time. The cells were harvested, and the phosphorylation of Chk1, KAP1, and H2AX was determined by Western blot analysis (*A* and *C*) and quantified by densitometry (*B* and *D*). GAPDH levels were determined to control for protein loading. For densitometry, all conditions were normalized to the 120-min DEA/NO condition, which was set at 100%. Results are representative (*A* and *C*) or the average ± SEM (*B* and *D*) of three independent experiments. Statistically significant differences between untreated and DEA/NO-treated groups are indicated (∗*p* < 0.05).ATM, ataxia–telangiectasia-mutated protein; ATR, ataxia–telangiectasia and Rad3-related protein; Chk1, checkpoint kinase 1; DDR, DNA damage response; DEA/NO, 2-(*N*,*N*-diethylamino)-diazenolate-2-oxide; H2AX, H2A histone family member X; KAP1, Krüppel-associated box–associated protein 1.

When inhibited, RNR causes replication stress resulting in SSBs and DDR activation (33, 34). While RNR was one of the first enzymes shown to be inhibited by nitric oxide (38, 39), the effects of this inhibition on ATR activation have yet to be examined. The quick-releasing nitric oxide donor 2-(*N*,*N*-diethylamino)-diazenolate-2-oxide (DEA/NO) (3 min *t*_1/2_) stimulates the formation of γH2AX and the phosphorylation of KAP1 and Chk1 in INS 832/13 cells and mouse embryonic fibroblasts (MEF) (Fig. 1). Temporally, phosphorylation of the ATR selective substrate Chk1 is observed 15 min after treatment in both cell types, and this is followed by γH2AX formation and KAP1 phosphorylation at later time points (60 min in INS 832/13 cells or 30 min in MEF). Since Chk1 is a selective substrate of ATR, these findings suggest that nitric oxide stimulates an early activation of ATR (15 min) in both cell types. ATR activation appears to be followed by a later activation of ATM (60 min in INS 832/13 cells and 30 min in MEF). This temporal difference is consistent with the inhibition of RNR by nitric oxide, whereas nitric oxide-induced ATM activation occurs in response to DNA double-strand break formation (11).

**Figure 2:**
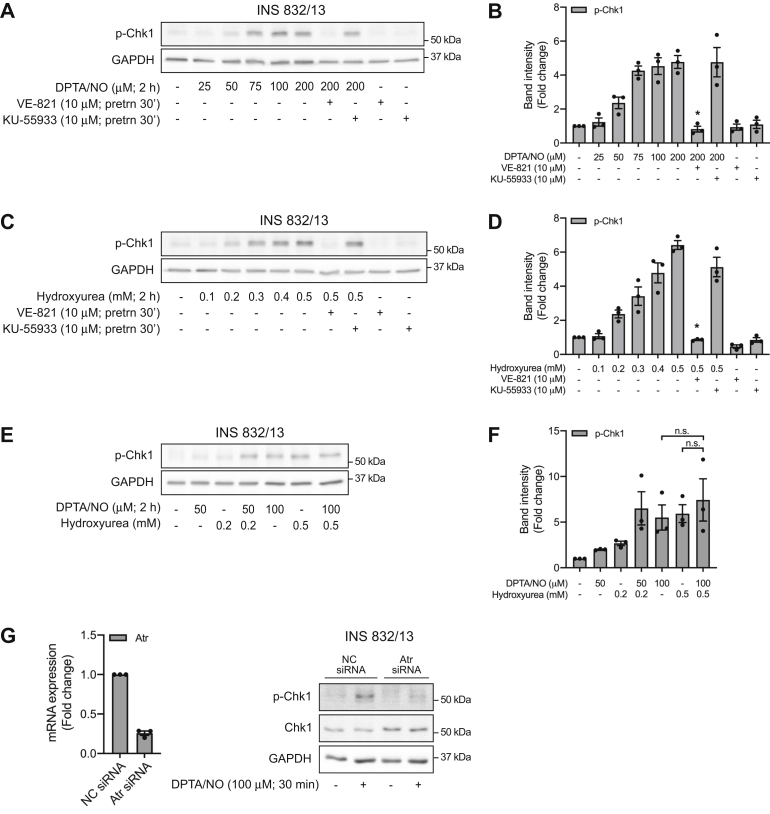
**Nitric oxide activates ATR signaling by inhibiting ribonucleotide reductase.** INS 832/13 cells (*A*–*D*) were treated with the indicated concentrations of DPTA/NO (*A* and *B*) or hydroxyurea (*C* and *D*) in the presence or absence of the ATR inhibitor VE-821 or the ATM inhibitor KU-55933 (30 min inhibitor pretreatment). Following an incubation of 2 h, the cells were harvested and the phosphorylation of Chk1 was determined by Western blot analysis (*A* and *C*) and quantified by densitometry (*B* and *D*). INS 832/13 cells (*E* and *F*) were treated with DPTA/NO and/or hydroxyurea at concentrations that induce half-maximal or maximal Chk1 phosphorylation following a 2-h exposure. The phosphorylation of Chk1 was determined by Western blot analysis (*E*) and quantified by densitometry (*F*). GAPDH levels were determined to control for protein loading. For densitometry, all conditions were normalized to the untreated group, which was set at one. INS 832/13 cells were transfected with either negative control (NC) siRNA or Atr siRNA for 48 h prior to 30-min DPTA/NO treatment (*G*). Knockdown efficiency was determined by quantitative RT-PCR and Chk1 phosphorylation and total Chk1 were determined by Western blot analysis. Results are representative (*A*, *C*, *E*, and *G*) or the average ± SEM (*B*, *D*, *F*, and *G*) of three independent experiments. Statistically significant inhibition of DPTA/NO- and hydroxyurea-induced Chk1 phosphorylation (*B* and *D*) are indicated (∗*p* < 0.05). ATR, ataxia–telangiectasia and Rad3-related protein; Chk1, checkpoint kinase 1; DPTA/NO, (Z)-1-[*N*-(3-aminopropyl)-*N*-(3-ammoniopropyl)amino]diazen-1-ium-1,2-diolate.

To further explore the temporal discordance in DDR kinase activation by nitric oxide, the effects of a second donor with a longer half-life (Z)-1-[*N*-(3-aminopropyl)-*N*-(3-ammoniopropyl)amino]diazen-1-ium-1,2-diolate (DPTA/NO; 3 h) that releases nitric oxide at more physiological relevant levels (1–5 μM) on ATR activation were examined (14). DPTA/NO stimulates Chk1 phosphorylation in INS 832/13 cells in a concentration-related manner that is first apparent at 50 μM and maximal at 100 μM (Fig. 2, *A* and *B*). Inhibition of ATR (VE-821) but not ATM (KU-55933) at a concentration we have shown to inhibit ATM signaling in INS 832/13 cells (11, 16) attenuates DPTA/NO-induced Chk1 phosphorylation (Fig. 2, *A* and *B*). The RNR inhibitor hydroxyurea also stimulates Chk1 phosphorylation in a concentration-dependent manner (Fig. 2, *C* and *D*) that is sensitive to ATR inhibition (VE-821) but not ATM inhibition (KU-55933). The inhibitory effects of VE-821 are concentration dependent with over a 80% inhibition at 1 μM and nearly complete inhibition of hydroxyurea-induced Chk1 phosphorylation at 10 μM (data not shown). These findings suggest that nitric oxide and hydroxyurea stimulate ATR activation by similar mechanisms. Consistent with this conclusion, the effects of submaximal concentrations of DPTA/NO (50 μM) and hydroxyurea (0.2 mM) on Chk1 phosphorylation are additive, while the response to maximally effective concentrations is not additive (Fig. 2, *E* and *F*). These experiments also suggest that Chk1 is an ATR selective substrate as inhibition and siRNA knockdown of this DDR kinase attenuates DPTA/NO-induced Chk1 phosphorylation in INS 832/13 cells (Fig. 2*G*).

### Nitric oxide as an inhibitor of ATR-dependent DDR signaling

**Figure 3:**
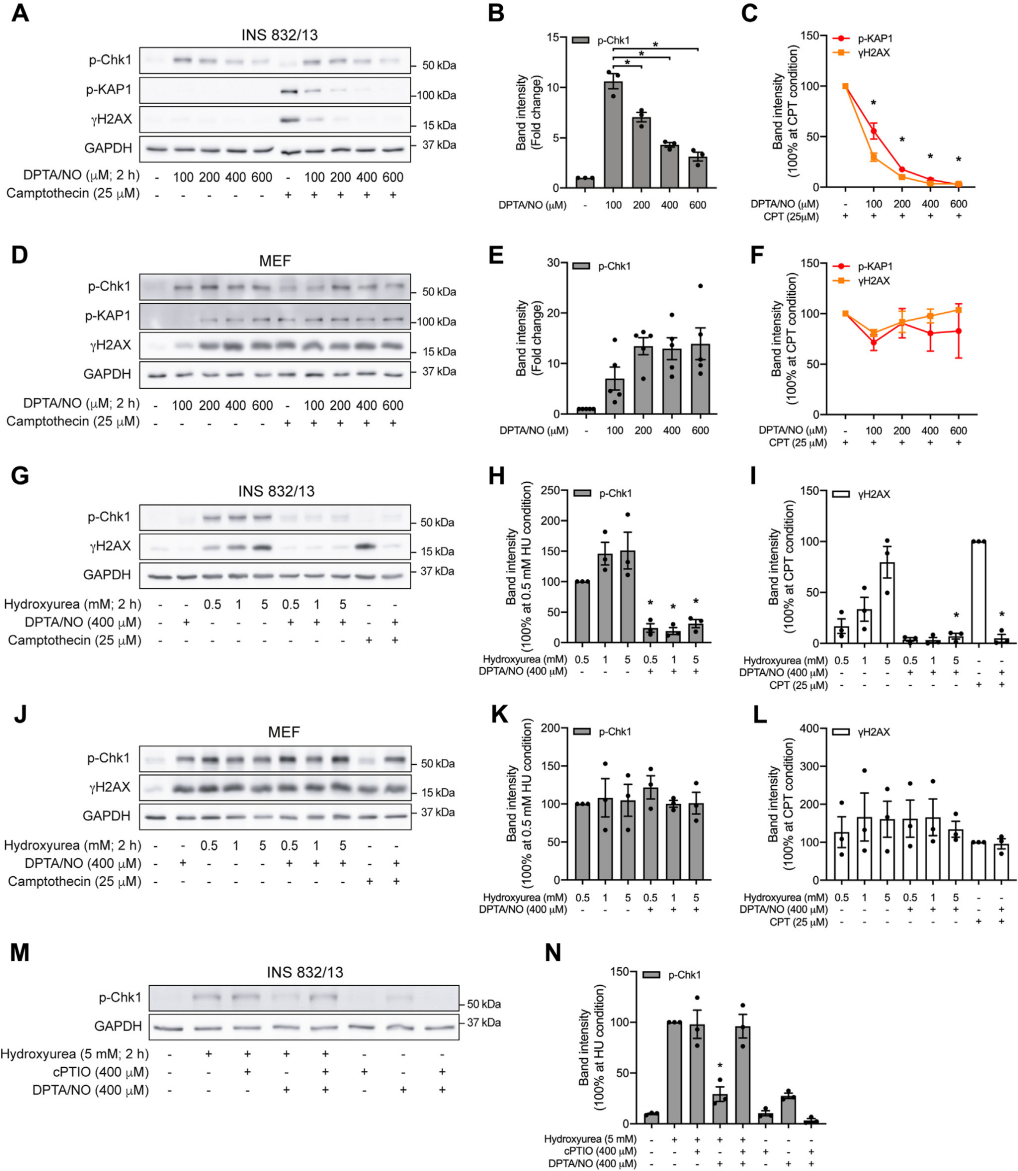
**Cell type selective inhibition of ATR signaling by nitric oxide**. INS 832/13 cells (*A*–*C* and *G*–*I*) and MEF (*D*–*F* and *J*–*L*) were treated with the indicated concentrations of DPTA/NO, hydroxyurea, or camptothecin for 2 h alone or in combination, or INS 832/13 cells (*M* and *N*) were treated with hydroxyurea in the presence or absence of cPTIO or DPTA/NO for 2 h. The phosphorylation of Chk1, KAP1, and H2AX was determined by Western blot analysis (*A*, *D*, *G*, *J*, and *M*) and quantified by densitometry (*B*, *C*, *E*, *F*, *H*, *I*, *K*, *L*, and *N*). GAPDH levels were determined to control for protein loading. For densitometry in *B* and *E*, all conditions were normalized to the untreated group and set at one; in *C*, *F*, *H*, *I*, *K*, *L*, and *N*, all conditions were normalized to hydroxyurea- or camptothecin-treated groups and set at 100%. Results are representative (*A*, *D*, *G*, *J*, and *M*) or the average ± SEM (*B*, *C*, *E*, *F*, *H*, *I*, *K*, *L*, and *N*) of two to five independent experiments. Statistically significant decrease in Chk1 phosphorylation (*B*) and inhibition of camptothecin-induced phosphorylation of KAP1 and H2AX (*C*), hydroxyurea-induced phosphorylation of Chk1 (*H* and *N*), and hydroxyurea- or camptothecin-induced γH2AX formation (*I*) are indicated (∗*p* < 0.05). ATR, ataxia–telangiectasia and Rad3-related protein; Chk1, checkpoint kinase 1; cPTIO, 2-(4-carboxyphenyl)-4,5-dihydro-4,4,5,5-tetramethyl-1H-imidazolyl-1-oxy-3-oxide; DPTA/NO, (Z)-1-[*N*-(3-aminopropyl)-*N*-(3-ammoniopropyl)amino]diazen-1-ium-1,2-diolate; H2AX, H2A histone family member X; KAP1, Krüppel-associated box–associated protein 1; MEF, mouse embryonic fibroblast.

In Figure 2, we show that the stimulatory effects of DPTA/NO on ATR-dependent Chk1 phosphorylation are concentration dependent. At 100 μM, DPTA/NO stimulates Chk1 phosphorylation in INS 832/13 cells (Fig. 3, *A* and *B*). As the concentration increases from 200 to 600 μM, the levels of Chk1 phosphorylation decrease to near basal levels (Fig. 3, *A* and *B*). Consistent with our previous studies (14, 16, 28), DPTA/NO alone does not stimulate γH2AX formation or KAP1 phosphorylation in INS 832/13 cells (Fig. 3*A*), yet inhibits these phosphorylation events in response to the DNA-damaging agent camptothecin (Fig. 3, *A* and *C*).

The inhibitory actions of nitric oxide on DDR signaling are selective for β-cells. DPTA/NO stimulates Chk1 phosphorylation in MEF at all concentrations examined (Fig. 3, *D* and *E*). Furthermore, DPTA/NO does not inhibit camptothecin-induced KAP1 phosphorylation and γH2AX formation in MEF (Fig. 3, *D* and *F*). In fact, DPTA/NO stimulates KAP1 phosphorylation and γH2AX formation in MEF (Fig. 3*D*). These findings are consistent with our previous studies showing that nitric oxide inhibits ATM-dependent DDR signaling selectively in β-cells (14, 16, 28).

Like ATM-dependent DDR signaling, nitric oxide also inhibits ATR signaling in a β-cell selective manner. In INS 832/13 cells and MEF, hydroxyurea stimulates the formation of γH2AX and Chk1 phosphorylation with maximal stimulation observed at 5 mM (Fig. 3, *G*–*L*). DPTA/NO at 400 μM attenuates hydroxyurea-induced Chk1 phosphorylation and γH2AX formation in INS 832/13 cells but not in MEF. As a positive control, we show that camptothecin-induced DDR signaling is also attenuated by DPTA/NO in INS 832/13 cells but not in MEF (Fig. 3, *G*–*L*). These findings provide evidence that nitric oxide inhibits ATR-dependent DDR signaling in a β-cell selective manner, similar to our previous findings for ATM-dependent DDR signaling (14, 16, 28).

The structure of hydroxyurea is similar to the *N*-hydroxyguanidine group of N^w^-hydroxy-l-arginine, an intermediate in the oxidation of arginine by NOS (40), and hydroxyurea releases a nitric oxide-like nitrosating reactant in the presence of hydrogen peroxide and copper(II) (41). The possibility that hydroxyurea may activate ATR by liberating nitric oxide or a nitrosating agent was examined by treating INS 832/13 cells with hydroxyurea in the presence or absence of the nitric oxide scavenger 2-(4-carboxyphenyl)-4,5-dihydro-4,4,5,5-tetramethyl-1H-imidazolyl-1-oxy-3-oxide (cPTIO). This nitric oxide scavenger attenuates DPTA/NO-induced Chk1 phosphorylation and the inhibitory actions of DPTA/NO on hydroxyurea-induced Chk1 phosphorylation but does not modify hydroxyurea-stimulated ATR activation (Fig. 3, *M* and *N*). These findings suggest that hydroxyurea does not activate ATR by liberating nitric oxide.

### Actions of endogenous iNOS-derived nitric oxide on DDR signaling

**Figure 4:**
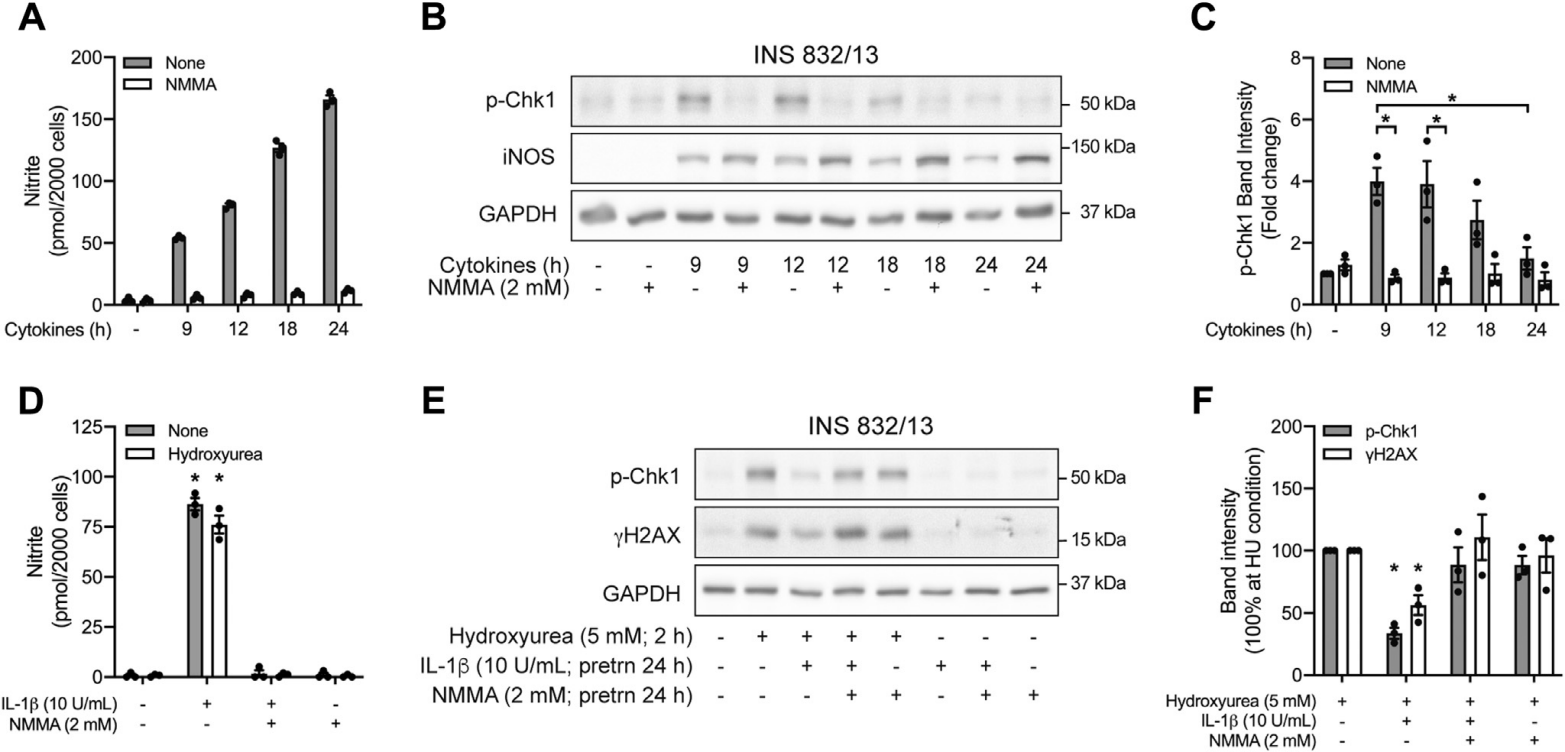
**Effects of endogenously derived nitric oxide on ATR signaling.** INS 832/13 cells were treated with cytokines (IL-1β, 10 U/ml and IFN-γ,150 U/ml) with or without 2 mM NMMA for the indicated times. Nitrite formation was determined using the culture supernatants (*A*) and the phosphorylation of Chk1 and iNOS was determined by Western blot analysis (*B*) and quantified by densitometry (*C*) where the untreated group was set at one. INS 832/13 cells were pretreated for 24 h with IL-1β with or without NMMA, hydroxyurea was added, and the cells were cultured for two additional hours. Nitrite formation was determined in the culture supernatants (*D*), and the phosphorylation of Chk1 and H2AX was determined by Western blot analysis (*E*) and quantified by densitometry (*F*) where all conditions were normalized to hydroxyurea-treated group, which was set at 100%. GAPDH levels were determined to control for protein loading. Results are representative (*B* and *E*) or the average ± SEM (*A*, *C*, *D*, and *F*) of three independent experiments. Statistically significant decrease in Chk1 phosphorylation at 24-h cytokine treatment (*C*), inhibition of cytokine-induced Chk1 phosphorylation by NMMA (*C*), IL-1β-induced nitrite formation (*D*), and inhibition of hydroxyurea-induced phosphorylation of Chk1 and H2AX by IL-1β (*F*) are indicated (∗*p* < 0.05). ATR, ataxia–telangiectasia and Rad3-related protein; Chk1, checkpoint kinase 1; IFN-γ, interferon gamma; IL-1β, interleukin-1β; iNOS, inducible nitric oxide synthase; NMMA, N^G^-monomethyl-l-arginine.

Cytokines (IL-1β + interferon-γ (IFN-γ)) stimulate iNOS expression and the time-dependent production of nitric oxide by INS 832/13 cells (Fig. 4, *A* and *B*). Early in this response (following incubations of 9 and 12 h), Chk1 is phosphorylated in a nitric oxide-dependent manner as it is sensitive to iNOS inhibition by N^G^-monomethyl-l-arginine (NMMA) (Fig. 4, *A*–*C*). Following longer exposures of 18 to 24 h, cytokines produce higher levels of nitric oxide (Fig. 4*A*), and Chk1 phosphorylation is no longer detected (Fig. 4, *B* and *C*). These findings are consistent with the concentration-dependent effect of DPTA/NO on ATR signaling where low concentrations stimulate, whereas higher concentrations fail to induce Chk1 phosphorylation (Fig. 3, *A* and *B*).

To determine if the endogenous production of nitric oxide is sufficient to inhibit ATR signaling, INS 832/13 cells were treated for 24 h with IL-1β in the presence or absence of NMMA. Hydroxyurea was then added, and ATR signaling was examined following an additional 2 h incubation. As shown in Figure 4 *D*–*F*, the stimulatory actions of hydroxyurea on Chk1 phosphorylation and γH2AX formation are attenuated in cells treated with IL-1β, and this effect is prevented by inhibiting the endogenous production of nitric oxide using NMMA. Alone, NMMA does not modify the INS 832/13 cell response to hydroxyurea.

**Figure 5:**
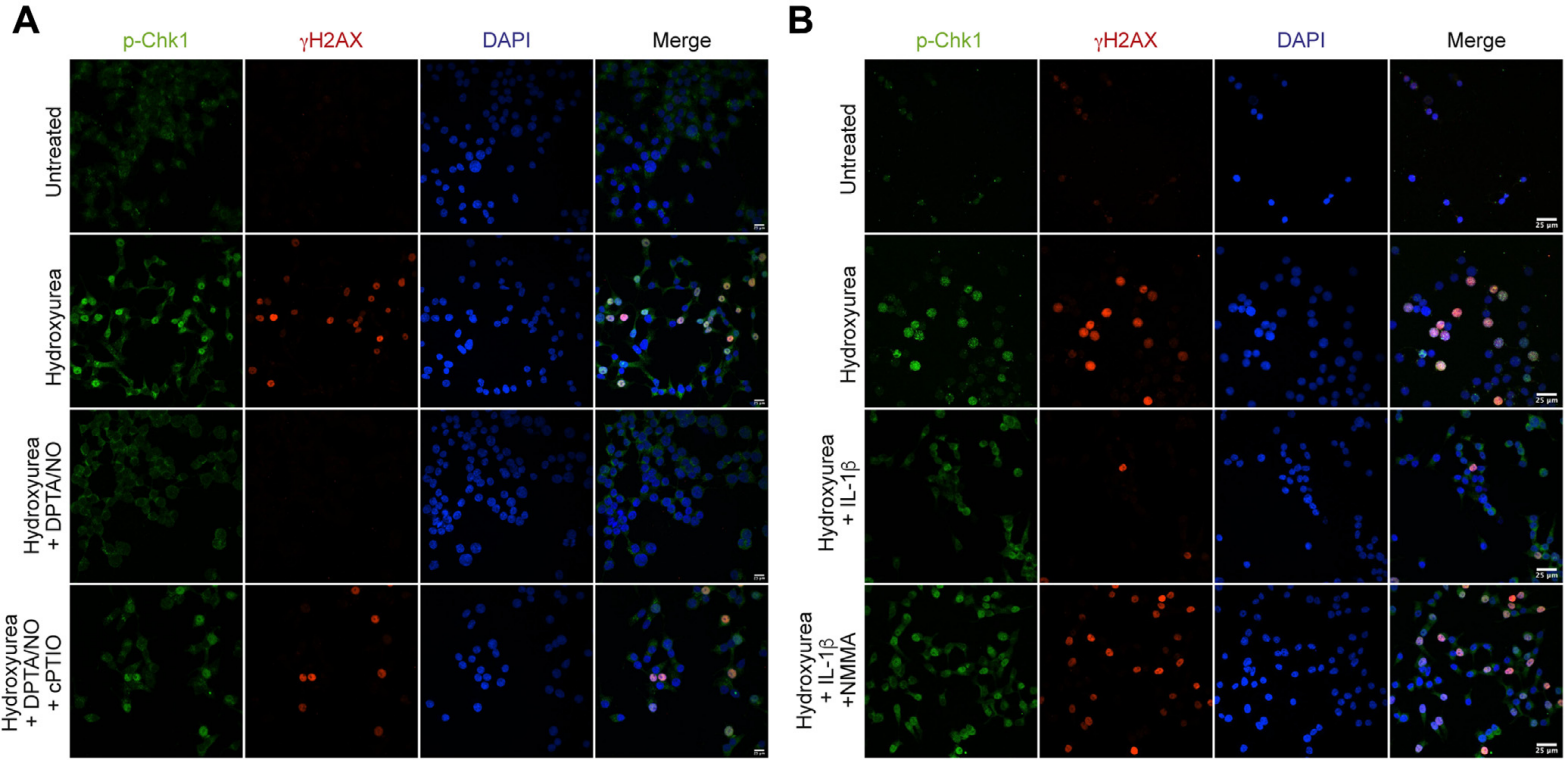
**Nitric oxide inhibits ATR-dependent DDR signaling in INS 832/13 cells.** INS 832/13 cells were treated with 5 mM hydroxyurea for 2 h in the presence or absence of 400 μM DPTA/NO and 400 μM cPTIO (*A*), or hydroxyurea was added after a 24-h incubation with 10 U/ml IL-1β in the presence or absence of 2 mM NMMA (*B*). Cells were then stained for phospho-Chk1 (*green*), γH2AX formation (*red*), and nuclei (DAPI, *blue*) and imaged *via* Nikon Eclipse 90i confocal microscope (60× with 2× field zoom). Results are representative of three independent experiments. ATR, ataxia–telangiectasia and Rad3-related protein; cPTIO, 2-(4-carboxyphenyl)-4,5-dihydro-4,4,5,5-tetramethyl-1H-imidazolyl-1-oxy-3-oxide; DDR, DNA damage response; DPTA/NO, (Z)-1-[*N*-(3-aminopropyl)-*N*-(3-ammoniopropyl)amino]diazen-1-ium-1,2-diolate; NMMA, N^G^-monomethyl-l-arginine; DAPI, 4′,6-diamidino-2-phenylindole.

Using a similar experimental design, the effects of nitric oxide on DDR signaling were examined by immunofluorescence microscopy. Hydroxyurea stimulates the nuclear localization of phosphorylated Chk1 and γH2AX in INS 832/13 cells (Fig. 5). Consistent with our biochemical approaches, nuclear localization of phosphorylated Chk1 and γH2AX is inhibited by nitric oxide supplied exogenously using 400 μM DPTA/NO (Fig. 5*A*) or produced endogenously following a 24 h incubation with IL-1β (Fig. 5*B*). As controls, we show that the inhibition of DDR signaling by nitric oxide is attenuated by cPTIO and NMMA (Fig. 5).

### Metabolic effects of nitric oxide

**Figure 6:**
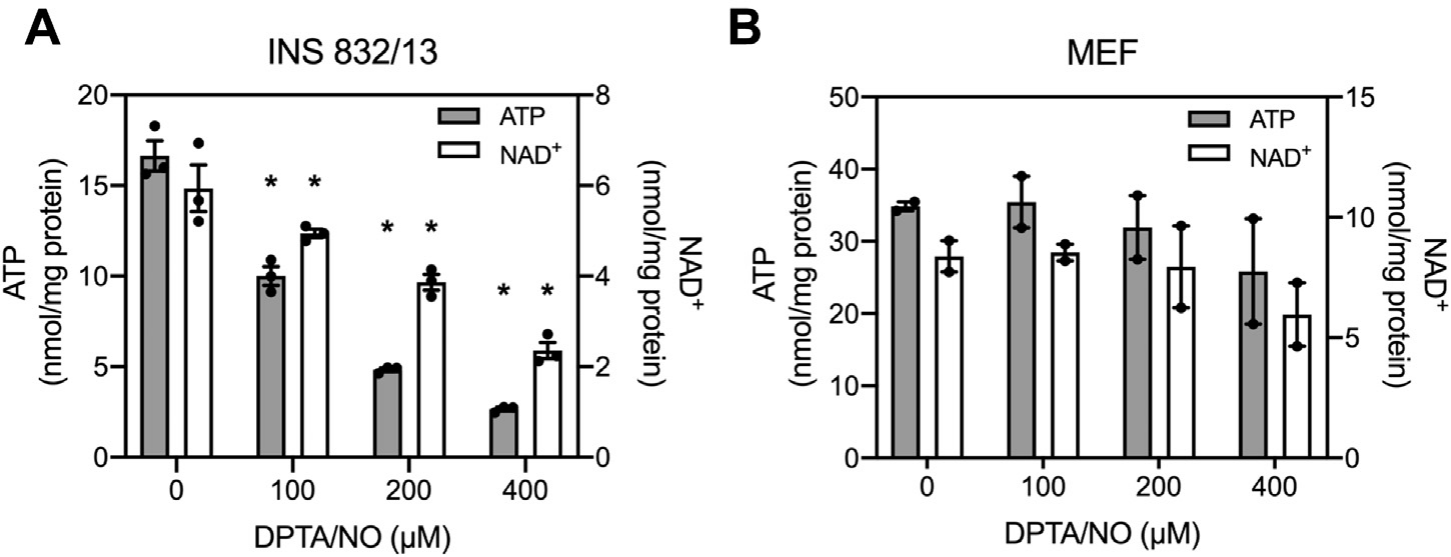
**Effects of nitric oxide on ATP and NAD**^**+**^**levels.** INS 832/13 cells (*A*) and MEF (*B*) were treated with DPTA/NO at the indicated concentrations for 2 h and then ATP (*gray bars*) and NAD^+^ (*white bars*) levels were determined. Results are the average ± SEM of two to three independent experiments. Statistically significant differences between untreated and DPTA/NO-treated groups are indicated (∗*p* < 0.05). DPTA/NO, (Z)-1-[*N*-(3-aminopropyl)-*N*-(3-ammoniopropyl)amino]diazen-1-ium-1,2-diolate; MEF, mouse embryonic fibroblast.

Essential for glucose-induced insulin secretion is the coupling of glycolysis with mitochondrial oxidative metabolism (18, 19). Nitric oxide inhibits aconitase of the Krebs cycle and complex IV of the electron transport chain (26, 27) resulting in a decrease in β-cell ATP levels by five- to ten-fold (28, 29, 30, 31). Consistent with these previous findings, DPTA/NO decreases INS 832/13 cell ATP in a concentration-dependent manner with a maximal effect observed at 400 μM (Fig. 6*A*; *gray bars*). The loss in ATP is associated with a similar decrease in NAD^+^ (Fig. 6*A*; *white bars*). Unlike in β-cells, DPTA/NO does not significantly decrease ATP or NAD^+^ levels in MEF (Fig. 6*B*).

**Figure 7:**
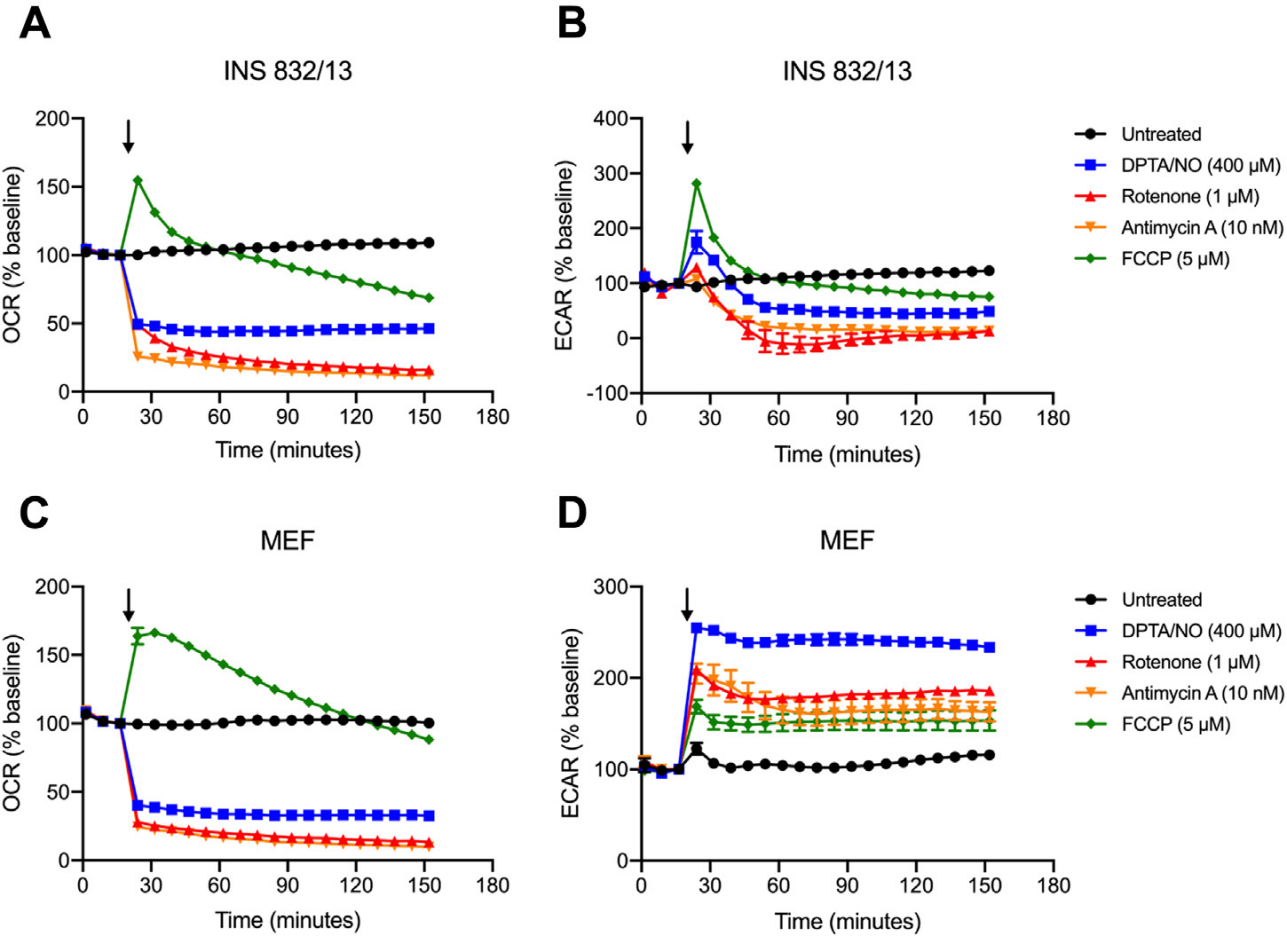
**Extracellular flux analysis of INS 832/13 cells and MEF.** The oxygen consumption rate (OCR) and extracellular acidification rate (ECAR) of INS 832/13 cells (*A* and *B*) and MEF (*C* and *D*) were measured over a 2-h incubation. The time of DPTA/NO (400 μM), rotenone (1 μM), antimycin A (10 nM), or FCCP (5 μM) addition is indicated by the *black arrows*. Results are the average ± SEM of three independent experiments. DPTA/NO, (Z)-1-[*N*-(3-aminopropyl)-*N*-(3-ammoniopropyl)amino]diazen-1-ium-1,2-diolate; FCCP, carbonyl cyanide 4-(trifluoromethoxy)phenylhydrazone; MEF, mouse embryonic fibroblast.

Most cell types compensate for impaired mitochondrial oxidative metabolism with an increase in glycolytic flux that is made possible by the regeneration of NAD^+^ (42, 43) by lactate dehydrogenase (LDH). NAD^+^ is a cofactor of the glycolytic enzyme GAPDH (43) and is required for continued glycolysis and ATP generation under anaerobic conditions (44). Pancreatic β-cells express low levels of LDH (17, 28) and are not capable of compensating for impaired mitochondrial oxidative metabolism with an increase in glycolytic flux. Using extracellular flux analysis, we showed that DPTA/NO decreases the rate of oxygen consumption (OCR) in both INS 832/13 cells and MEF (Fig. 7, *A* and *C*). MEF compensates for impaired OCR with an increase in the extracellular acidification rate (ECAR), an indicator of glycolysis (Fig. 7*D*). INS 832/13 cells lack this metabolic flexibility as ECAR is not increased following DPTA/NO treatment (Fig. 7*B*).

### Mitochondrial respiratory inhibitors attenuate ATR signaling selectively in insulinoma cells

Like the action of nitric oxide, OCR is decreased by mitochondrial respiratory inhibitors rotenone (complex I) and antimycin A (complex III) in MEF and INS 832/13 cells (Fig. 7). MEF respond to this inhibition with an increase in glycolytic flux (ECAR), whereas INS 832/13 cells lack the metabolic flexibility to increase ECAR (Fig. 7, *B* and *D*). Carbonyl cyanide 4-(trifluoromethoxy)phenylhydrazone (FCCP) is a protonophore that uncouples ATP synthesis from electron transport and allows for the measurement of the maximal rate of oxygen consumption by cells (45). FCCP increases the OCR in both INS 832/13 cells and MEF (Fig. 7, *A* and *C*), and consistent with an uncoupling of ATP synthesis from the electron transport chain, it also stimulates a sustained increase in ECAR in MEF (Fig. 7*D*). Even though FCCP increases ECAR in INS 832/13 cells, it is not sustained and rapidly falls below basal levels (Fig. 7*B*).

**Figure 8:**
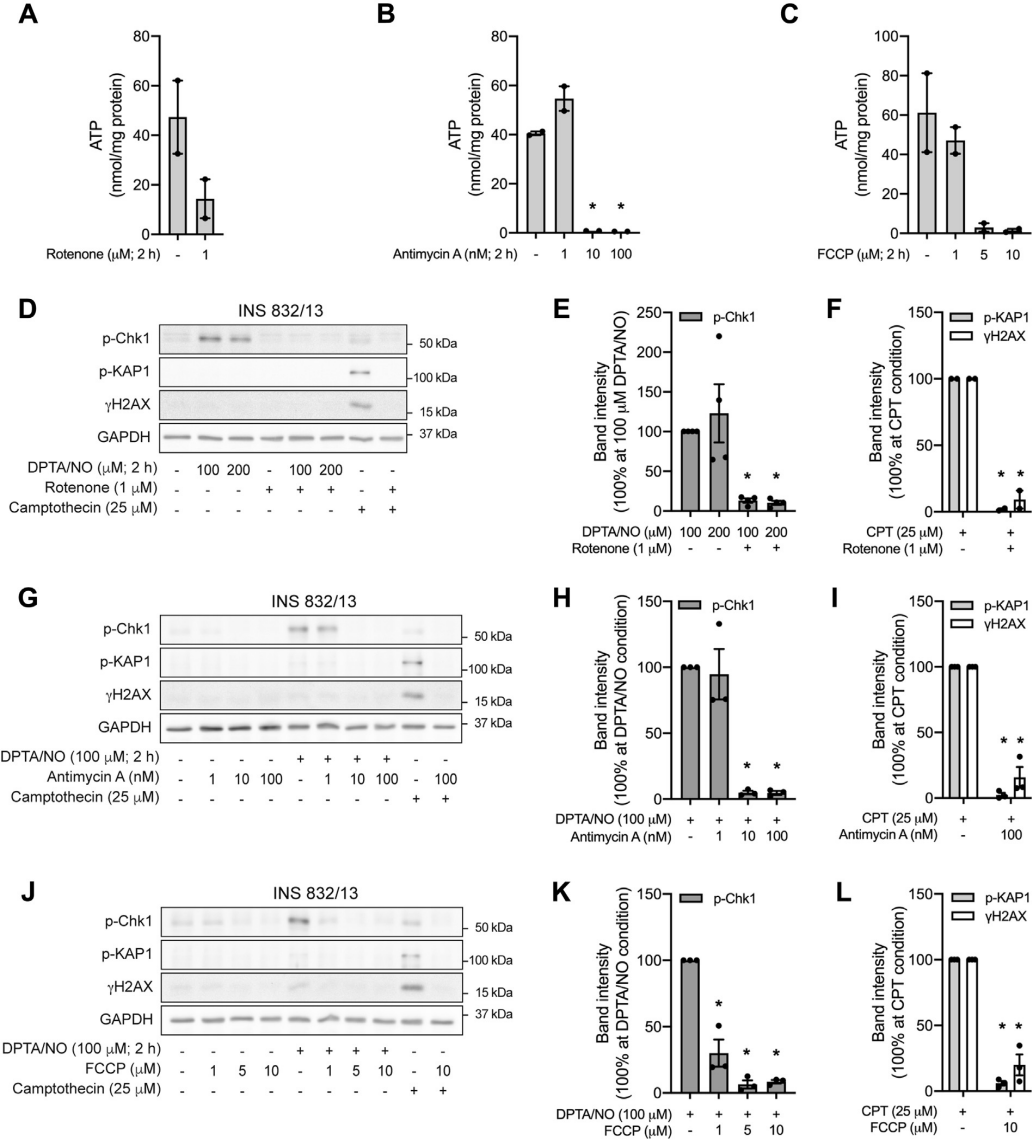
**Inhibitors of mitochondrial respiration attenuate ATR signaling.** INS 832/13 cells were treated with rotenone (*A*), antimycin A (*B*), or FCCP (*C*) for 2 h and then ATP levels were determined. INS 832/13 cells (*D*–*L*) were treated with DPTA/NO or camptothecin for 2 h, in the presence or absence of rotenone (*D*–*F*), antimycin A (*G*–*I*), or FCCP (*J*–*L*), as indicated. The phosphorylation of Chk1, KAP1, and H2AX was determined by Western blot analysis (*D*, *G*, and *J*) and quantified by densitometry (*E*, *F*, *H*, *I*, *K*, and *L*) where all conditions were normalized to DPTA/NO- or camptothecin-treated groups. GAPDH was determined to control for protein loading. Results are representative (*D*, *G*, and *J*) or the average ± SEM (*A*–*C*, *E*, *F*, *H*, *I*, *K*, and *L*) of two to four independent experiments. Statistically significant decreases in ATP and DDR signaling are indicated (∗*p* < 0.05). ATR, ataxia–telangiectasia and Rad3-related protein; Chk1, checkpoint kinase 1; DDR, DNA damage response; DPTA/NO, (Z)-1-[*N*-(3-aminopropyl)-*N*-(3-ammoniopropyl)amino]diazen-1-ium-1,2-diolate; FCCP, carbonyl cyanide 4-(trifluoromethoxy)phenylhydrazone; H2AX, H2A histone family member X; KAP1, Krüppel-associated box–associated protein 1.

We hypothesized that if the inhibition of mitochondrial oxidative metabolism and the lack of metabolic flexibility are responsible for the β-cell selective inhibition of ATR signaling by nitric oxide, then mitochondrial respiratory inhibitors should also attenuate ATR signaling. Consistent with this hypothesis, all three mitochondrial respiratory inhibitors (rotenone, antimycin A, and FCCP) attenuate DPTA/NO (100 or 200 μM)–stimulated Chk1 phosphorylation (Fig. 8, *D*, *E*, *G*, *H*, *J*, and *K*), and this inhibition is associated with decreases in INS 832/13 cell ATP levels (Fig. 8, *A*–*C*). As a control, we also show that the mitochondrial respiratory inhibitors attenuate camptothecin-induced KAP1 phosphorylation and γH2AX formation (Fig. 8, *F*, *I*, and *L*) in a manner similar to our previous studies (28). These findings suggest that inhibition of mitochondrial oxidative metabolism is one mechanism by which nitric oxide impairs ATR signaling selectively in β-cells.

### ATR signaling in MEF lacking metabolic flexibility

**Figure 9:**
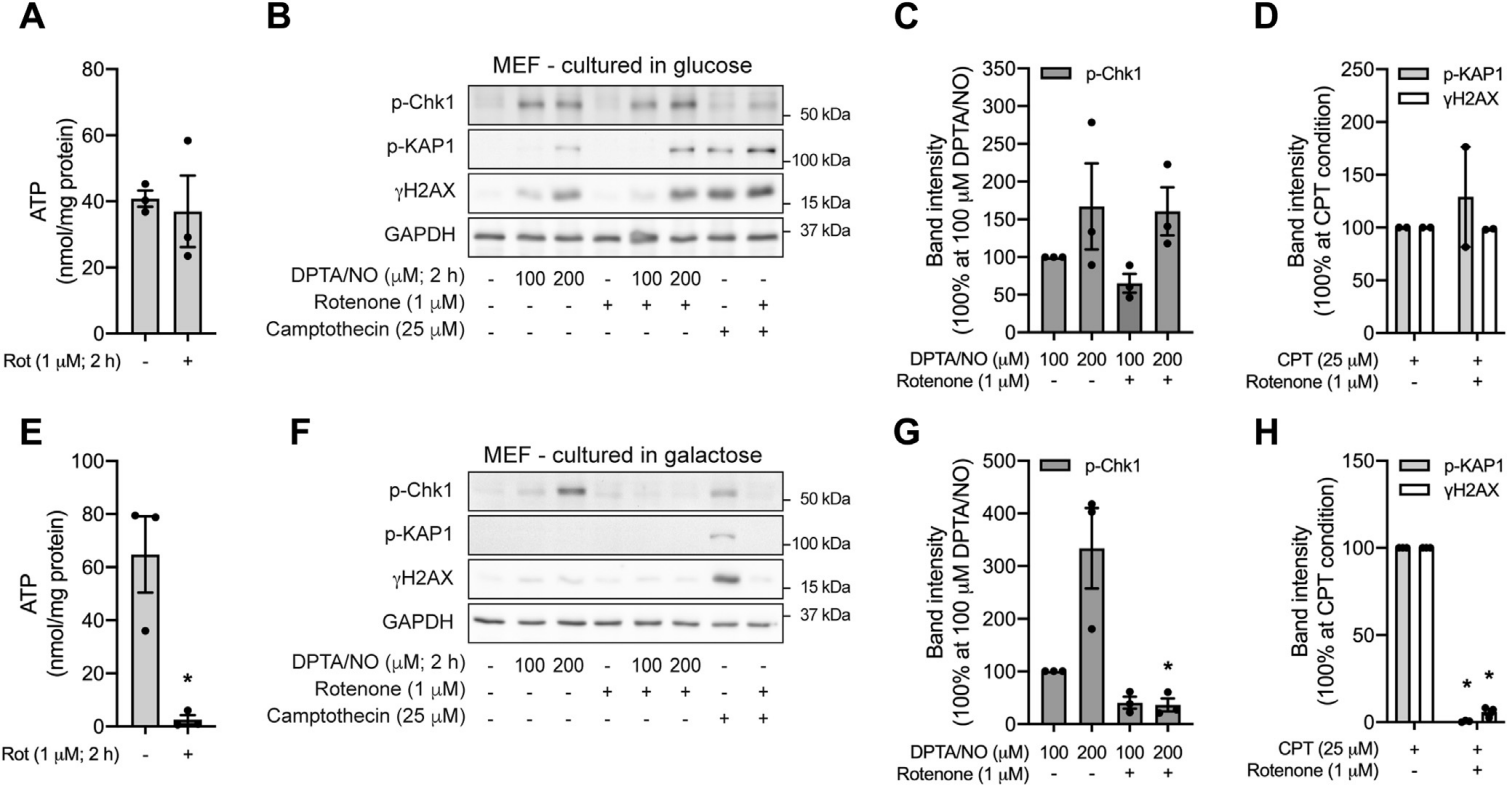
**The role of metabolic flexibility in the regulation of ATR signaling.** MEF, cultured in medium containing either glucose (*A*–*D*) or galactose (*E*–*H*) were treated for 2 h with DPTA/NO or camptothecin in the presence or absence of rotenone, as indicated. ATP levels were determined (*A* and *E*). The phosphorylation of Chk1, KAP1, and H2AX was determined by Western blot analysis (*B* and *F*) and quantified by densitometry (*C*, *D*, *G*, and *H*) where all conditions were normalized to DPTA/NO- or camptothecin-treated groups. GAPDH levels were determined to control for protein loading. Results are representative (*B* and *F*) or the average ± SEM (*A*, *C*, *D*, *E*, *G*, and *H*) of two to three independent experiments. Statistically significant decreases in ATP and the phosphorylation of DDR signaling are indicated (∗*p* < 0.05). ATR, ataxia–telangiectasia and Rad3-related protein; Chk1, checkpoint kinase 1; DDR, DNA damage response; DPTA/NO, (Z)-1-[*N*-(3-aminopropyl)-*N*-(3-ammoniopropyl)amino]diazen-1-ium-1,2-diolate; H2AX, H2A histone family member X; KAP1, Krüppel-associated box–associated protein 1; MEF, mouse embryonic fibroblast.

It is possible to force non–β-cells to generate ATP *via* mitochondrial oxidative metabolism by culturing in glucose-free medium containing galactose as the primary carbon source. Galactose is a poor substrate for glycolysis, and cells cultured in this medium generate ATP *via* the mitochondrial oxidation of glutamine (46, 47, 48). In MEF cultured in standard glucose-containing medium, the complex I inhibitor rotenone does not modify ATP levels or DPTA/NO (100 or 200 μM)–induced ATR activation as evidenced by Chk1 phosphorylation (Fig. 9, *A*–*C*). In contrast, when MEF are cultured in galactose-containing medium, these response mimics β-cells, as rotenone decreases ATP levels and inhibits DPTA/NO-induced ATR activation (Chk1 phosphorylation; Fig. 9, *E*–*G*). Furthermore, DPTA/NO fails to stimulate KAP1 phosphorylation or γH2AX formation in MEF cultured in galactose as compared with the stimulatory actions in glucose-containing medium, consistent with what we observe for INS 832/13 cells (Figs. 3 and 9). As controls, we show that rotenone inhibits camptothecin-stimulated Chk1 and KAP1 phosphorylation and γH2AX formation in galactose-cultured but not glucose-cultured MEF (Fig. 9, *D* and *H*). These findings provide additional evidence that the lack of metabolic flexibility and decreases in ATP are one mechanism by which the inhibition of mitochondrial oxidative metabolism (by nitric oxide and mitochondrial respiratory inhibitors) attenuates DDR signaling (both ATR and ATM) selectively in β-cells.

### Regulation of ATR signaling in islets

**Figure 10:**
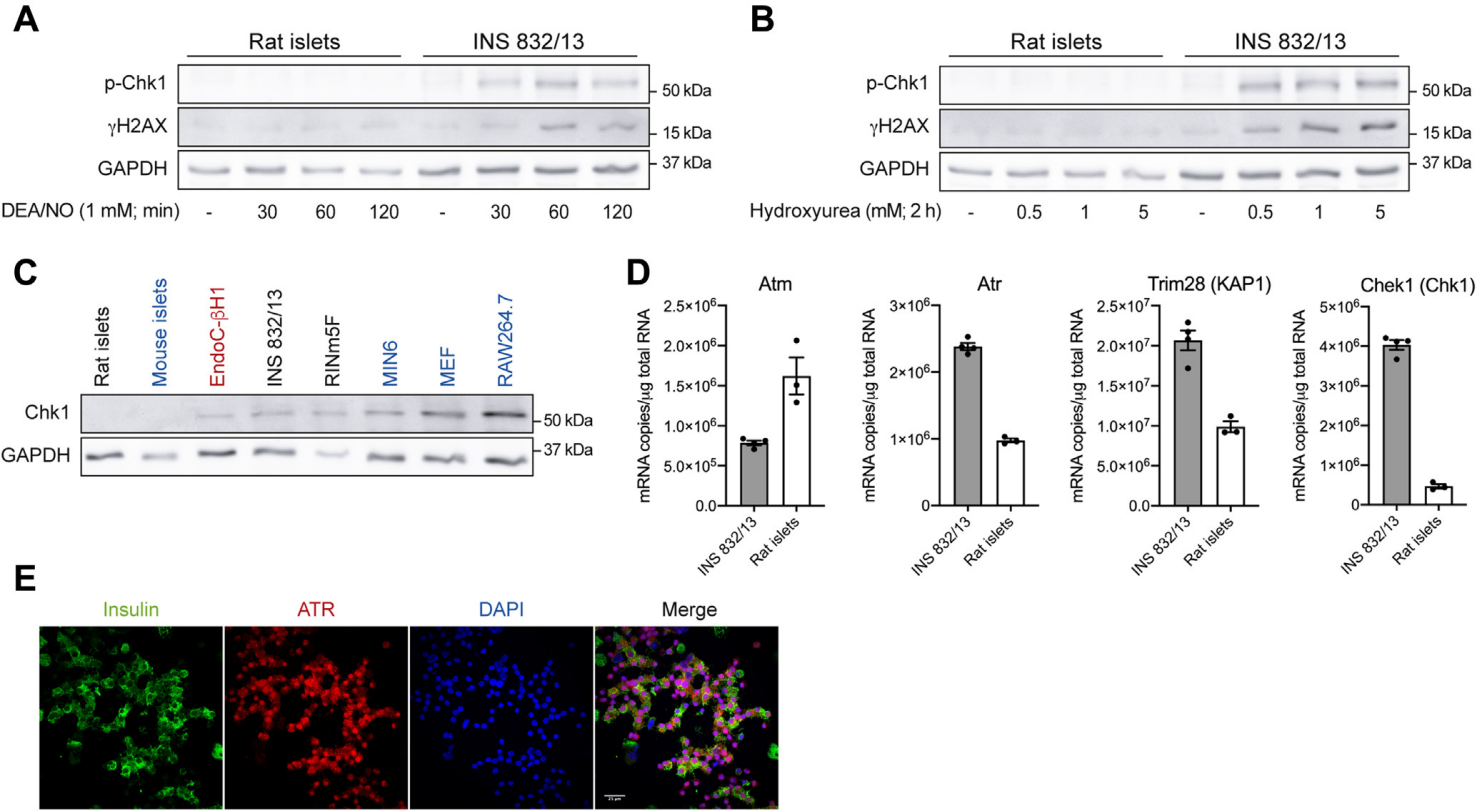
**Effects of nitric oxide on ATR signaling in islets.** Rat islets and INS 832/13 cells (*A* and *B*) were treated with DEA/NO for the indicated times (*A*) or treated for 2 h with hydroxyurea (*B*). The cells were harvested, and the phosphorylation of Chk1 and H2AX was determined by Western blot. GAPDH levels were determined to control for protein loading. The levels of Chk1 in rodent islets and various immortalized β-cell lines (EndoC-βH1, INS 832/13, RINm5F, and MIN6) and non–β-cell lines (MEF and RAW 264.7) were determined by Western blot analysis (*C*). The steady state levels of Atm, Atr, Trim28, and Chek1 mRNA in INS 832/13 cells and rat islets were quantified by quantitative RT-PCR (*D*). Islet cell expression of ATR was determined by immunofluorescence (*E*) of dispersed rat islet cells stained for insulin (*green*), ATR (*red*), and nuclei (DAPI, *blue*). Cells were visualized using Nikon Eclipse Ti2-E microscope equipped with a Yokogawa confocal scanner unit (CSU-W1) (60× with 2× field zoom). Results are representative (*A*, *B*, *C*, and *E*) or the average ± SEM (*D*) of two to four independent experiments. ATR, ataxia–telangiectasia and Rad3-related protein; Chk1, checkpoint kinase 1; DAPI, 4′,6-diamidino-2-phenylindole; DEA/NO, 2-(*N*,*N*-diethylamino)-diazenolate-2-oxide; H2AX, H2A histone family member X; MEF, mouse embryonic fibroblast.

DEA/NO and hydroxyurea induce Chk1 phosphorylation in INS 832/13 cells, whereas these ATR activators fail to induce Chk1 phosphorylation in rat islets (Fig. 10, *A* and *B*). The lack of ATR signaling in islets reflects low levels of Chk1 expression, as the steady state levels are below the limits of detection in rodent islets but are readily detectable in immortalized β-cell lines (EndoC-βH1, INS 832/13, RINm5F, and MIN6 cells) and non–β-cell lines such as MEF and RAW 264.7 macrophages by Western blot analysis (Fig. 10*C*). The steady state levels of Chek1 (gene name for Chk1) mRNA are much lower in rat islets than the levels found in INS 832/13 cells. Atm is highly expressed, and Atr and Trim28 (gene name for KAP1) are moderately expressed in rat islets as compared with the steadystate mRNA levels found in INS 832/13 cells (Fig. 10*D*). RNA-Seq data generated from fluorescence-activated cell sorting–purified mouse β-cells also showed that β-cells express very low levels of Chek1 (49). Chk1 is a threonine/serine protein kinase that mediates cell cycle arrest in response to DNA damage (6). The absence of Chk1 expression in islets is consistent with the low replication rates of β-cells, and studies suggest that replication interferes with insulin secretion (50). Although we have yet to identify additional substrates that are selective for ATR, this DDR kinase is expressed in islets and insulin-containing β-cells (Fig. 10, *D* and *E*), suggesting that ATR likely plays some physiological role.

## Discussion

We have previously shown that nitric oxide functions as both an activator and an inhibitor of the DDR kinase ATM (11, 14). When produced at low micromolar levels following iNOS expression, nitric oxide activates ATM by inducing DNA strand breaks (14). Nonetheless, the phosphorylation of ATM substrates KAP1 and H2AX are temporally dissociated from DNA damage (14). For example, treatment with a short half-life nitric oxide donor (DEA/NO, *t*_1/2_ = 3 min) results in a four-fold increase in DNA damage within 15 min of treatment, but KAP1 phosphorylation and γH2AX formation require a longer exposure of 60 to 90 min, or conditions in which the donor no longer releases nitric oxide at low micromolar iNOS-derived levels (14). Furthermore, when actively produced at low micromolar levels exogenously using donors or endogenously following iNOS expression, nitric oxide attenuates hydrogen peroxide–, camptothecin-, and nitric oxide-induced ATM activation (14). The inhibitory actions on ATM activation are selective for pancreatic β-cells as nitric oxide does not inhibit but stimulates ATM activation in non–β-cells (14, 16, 28).

In the current study, the effects of nitric oxide on the activity of a second DDR transducer kinase, ATR, were examined. ATR is activated in response to SSBs and replication stress (33, 34). We show that low concentrations (50–200 μM) of a long half-life nitric oxide donor (DPTA/NO; Fig. 2) or short exposures to a short half-life nitric oxide donor (DEA/NO; Fig. 1) activate ATR signaling as evidenced by Chk1 phosphorylation. Much like the ATR activator hydroxyurea, nitric oxide-stimulated Chk1 phosphorylation is sensitive to ATR but not ATM inhibition (Fig. 2). The activation of ATR is not cell type selective, as nitric oxide stimulates Chk1 phosphorylation in both MEF and insulinoma cells (Figs. 2 and 3). While these findings provide the first evidence that nitric oxide activates ATR signaling, they are not surprising as nitric oxide is known to inhibit RNR (38, 39). Hydroxyurea activates ATR signaling by quenching the tyrosyl radical at active site of RNR (51). While hydroxyurea has been shown to release a nitrosating agent that may target this tyrosyl radical (41), scavenging nitric oxide using cPTIO does not modify hydroxyurea-induced Chk1 phosphorylation in INS 832/13 cells (Fig. 3). These findings support the inhibition of RNR as the mechanism by which nitric oxide activates ATR signaling and that hydroxyurea does not activate ATR by releasing nitric oxide.

As the concentration of DPTA/NO increases to 400 μM, nitric oxide no longer activates ATR but inhibits hydroxyurea-induced ATR activation (Fig. 3). Also, when endogenously produced following cytokine-stimulated iNOS expression, nitric oxide activates ATR signaling following 9 and 12 h treatments but inhibits ATR signaling following 18 and 24 h incubation or conditions in which there is an increase in the amount of nitric oxide produced (Fig. 4). These findings suggest that there is a threshold concentration where nitric oxide functions as an activator of ATR, and when produced at levels above this threshold, nitric oxide inhibits ATR-dependent signaling. For DPTA/NO, the threshold is ∼200 μM, where the competing actions of nitric oxide on ATR activation (because of the inhibition of RNR) and inhibition are balanced or near equilibrium.

The inhibitory actions of nitric oxide on ATR signaling are selective for β-cells (Fig. 3). This finding is similar to the β-cell selective inhibitory actions of nitric oxide on ATM signaling (14). The selective inhibition of DDR signaling in β-cells is associated with differences in the regulation of intermediary metabolism between β-cells and most non–β-cell types. The coupling of glycolysis and mitochondrial oxidative metabolism is essential for glucose-stimulated insulin secretion (17, 18, 19, 20, 21). In non–β-cells, mitochondrial oxidation occurs on demand for ATP or products of anaplerotic reactions (42). Under anaerobic conditions or impaired mitochondrial metabolism, non–β-cells compensate with an increase in glycolysis, using LDH to regenerate NAD^+^ (42). β-cells lack this metabolic flexibility as they express low levels of LDH and are not capable of replenishing NAD^+^ in response to oxygen deprivation or inhibitors of mitochondrial respiration (17, 28). As an inhibitor of aconitase and complex IV of the electron transport chain (26, 27), nitric oxide depletes β-cell ATP, whereas non–β-cells maintain ATP levels by increasing glycolytic flux (17, 28).

Consistent with this mechanism of action, mitochondrial respiratory inhibitors (rotenone, antimycin A, and FCCP) decrease ATP levels and attenuate ATR-dependent DDR signaling selectively in β-cells (Fig. 8). Non–β-cells forced to generate ATP *via* mitochondrial metabolism (culturing in glucose-deficient galactose-containing medium) become sensitive to the inhibitory actions of mitochondrial respiratory inhibitors on ATR activation (Fig. 9). These findings suggest that the inhibition of mitochondrial oxidative metabolism and the lack of metabolic flexibility are responsible for the β-cell selective inhibitory actions of iNOS-derived low micromolar levels of nitric oxide on DDR signaling (ATM and ATR).

We were unable to confirm these findings in islets because of the low expression levels of the ATR substrate Chk1 and challenges in the identification of additional ATR selective substrates that are expressed in β-cells (Fig. 10) (49). Also, many of the known substrates can be phosphorylated by multiple DDR kinases. As an example, ATR has been shown to phosphorylate H2AX and p53 (9, 52); however, both substrates are phosphorylated by additional DDR kinases including ATM (53). Furthermore, we have shown that nitric oxide attenuates hydrogen peroxide–induced γH2AX formation in rat islets (14), and while hydrogen peroxide is an ATR activator (54), ATM is also activated by the DNA damage induced by this oxidant (53).

Cytokines are believed to contribute to β-cell destruction during the development of autoimmune diabetes (55, 56), as they have been shown to impair β-cell function and cause β-cell destruction in a nitric oxide-dependent manner (57, 58). Nitric oxide inhibits insulin secretion by attenuating mitochondrial oxidative metabolism and decreasing ATP (23, 24, 25). The damaging actions of cytokine-derived nitric oxide have been demonstrated in rodent and human islets of Langerhans, and many of these studies have led to the hypothesis that cytokines contribute to, or trigger, β-cell damage during the development of autoimmune diabetes (56, 59, 60).

**Figure 11:**
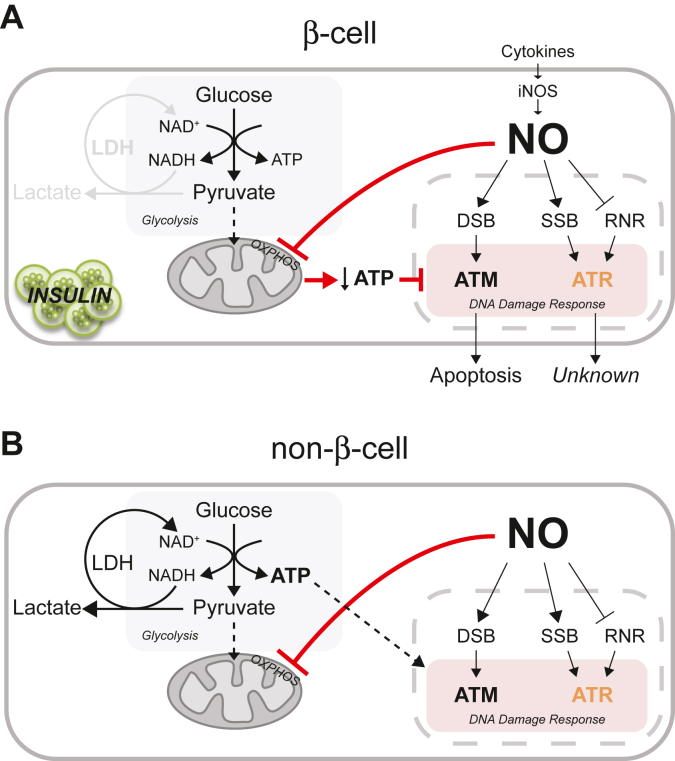
**Proposed mechanism for dual regulation of DDR signaling by nitric oxide in β-cells.** In response to cytokines, β-cells express iNOS and produce nitric oxide. Early in this process, nitric oxide inhibits RNR causing replication fork stress and ATR activation (*A* and *B*). As the treatment period is extended and the levels of nitric oxide increase, mitochondrial oxidative metabolism is inhibited (aconitase and complex IV of the electron transport chain). While most cell types (*B*) have the metabolic flexibility to increase glycolysis, β-cells (*A*) lack this flexibility and ATP decreases. It is the inhibition of mitochondrial oxidative metabolism and the resulting decrease in ATP that correlates with an inhibitory action of nitric oxide on ATM and ATR signaling in a β-cell selective manner. ATR, ataxia–telangiectasia and Rad3-related protein; DDR, DNA damage response; iNOS, inducible nitric oxide synthase; RNR, ribonucleotide reductase.

While this is an attractive hypothesis, we have shown that the inhibitory effects of cytokines on insulin secretion, oxidative metabolism, and protein synthesis are reversible (61, 62, 63) and that rodent and human β-cells are capable of repairing damaged DNA (64). Furthermore, nitric oxide is an effective inhibitor of caspase activity (65, 66), including DDR-directed β-cell apoptosis (14). We now show that nitric oxide activates ATR signaling by inhibiting RNR and that iNOS-derived low micromolar levels of nitric oxide attenuate ATR-dependent DDR signaling in a manner similar to the inhibition of ATM-dependent DDR signaling (14). Importantly, nitric oxide inhibits DDR signaling by targeting mitochondrial oxidative metabolism or the same pathways used by β-cells to regulate glucose-induced insulin secretion (Fig. 11). The reversibility of cytokine-mediated damage and the ability of nitric oxide to activate protective pathways (such as the unfolded protein and heat shock responses as well as DNA repair) suggest that there may be physiological roles for these responses. Consistent with this view, β-cells are terminally differentiated with a limited capacity to replicate (50), yet essential for survival as they are the only cell types capable of producing insulin. They are also responsive to a cytokine (IL-1) whose serum levels spike over 1000-fold during infection, suggesting that there are physiological roles for this responsiveness. While additional studies are required to more fully appreciate the roles of nitric oxide production in regulating DDR signaling in β-cells, our studies are beginning to shed light on the physiological roles for cytokine signaling, iNOS expression, and the production of nitric oxide by β-cells. One such role being the inhibition of picornavirus replication in a pancreatic β-cell selective manner (67, 68).

## Experimental procedures

### Cell lines, animals, and materials

INS 832/13 cells were obtained from Dr Christopher Newgard (Duke University). MEF were obtained from Dr Fumihiko Urano (Washington University). EndoC-βH1 cells were obtained from Dr Raphael Scharfmann (Paris Descartes University). RINm5F, MIN6, and RAW 264.7 cells were obtained from the Washington University Tissue Culture Support Center. Male Sprague–Dawley rats were purchased from Harlan. Male and female C57BL/6J mice were purchased from The Jackson Laboratory. Connaught Medical Research Laboratories 1066 medium, d-glucose, and β-mercaptoethanol were purchased from Thermo Fisher Scientific. RPMI1640 medium, Dulbecco's modified Eagle's medium (DMEM), trypsin (0.05% in 0.53 mM EDTA), l-glutamine, sodium pyruvate, HEPES, and penicillin–streptomycin were purchased from Corning. Fetal bovine serum (FBS) was purchased from HyClone. Recombinant human IL-1β and rat IFN-γ were purchased from PeproTech. DEA/NO and L-NMMA were purchased from Enzo Life Sciences. DPTA/NO, VE-821, and cPTIO were purchased from Cayman Chemical. Hydroxyurea, camptothecin, KU-55933, rotenone, antimycin A, FCCP, and d-galactose were purchased from MilliporeSigma. Primary and secondary antibodies used for Western blot and immunofluorescence were purchased as follows: mouse anti-γH2AX (Ser139) from MilliporeSigma; rabbit anti-phospho-KAP1 (Ser824) from Abcam; rabbit anti-ATR, rabbit anti-phospho-Chk1 (Ser345), and mouse anti-Chk1 from Cell Signaling Technology; rabbit anti-iNOS from Cayman Chemical; mouse anti-GAPDH from Thermo Fisher Scientific; guinea pig anti-insulin from DakoCytomation; horseradish peroxidase (HRP)-conjugated donkey anti-mouse, HRP-conjugated donkey anti-rabbit, Alexa Fluor 488–conjugated donkey anti-rabbit, Cy3-conjugated donkey anti-rabbit, and Cy3-conjugated donkey anti-mouse from Jackson ImmunoResearch Laboratories, Inc; and Alexa Fluor 488–conjugated donkey anti-guinea pig from Molecular Probes. Negative control and ATR-targeted siRNAs were purchased from Integrated DNA Technologies.

### Cell culture and islet isolation

INS 832/13 cells were cultured in RPMI1640 medium supplemented with 10% FBS, 1 mM pyruvate, 2 mM l-glutamine, 10 mM HEPES, and 50 μM β-mercaptoethanol (69). MEF were cultured in DMEM containing either 25 mM glucose or 10 mM d-galactose, with 10% FBS, 1 mM pyruvate, 2 mM l-glutamine, and 10 mM HEPES (28, 69). Other cell lines used (EndoC-βH1, RINm5F, MIN6, and RAW264.7 cells) were cultured as previously described without penicillin and streptomycin (68, 70, 71, 72). Islets were isolated from Sprague–Dawley rats and C57BL/6J mice and were prepared as previously described (73, 74, 75). Isolated islets were cultured in Connaught Medical Research Laboratories-1066 medium supplemented with 10% FBS, 2 mM l-glutamine, 100 U/ml penicillin, and 100 μg/ml streptomycin (11). Animal studies and care were approved by the Institutional Animal Care and Use Committees at the Medical College of Wisconsin (A3102-01).

### Western blot analysis

Western blot analysis was performed as previously described (76). Antibody dilutions were as follows: 1:1000 for anti-phospho-Chk1 (Ser345), anti-Chk1, and anti-iNOS; 1:2000 for anti-phospho-KAP1 (Ser824); 1:10,000 for anti-γH2AX (Ser139); 1:20,000 for anti-GAPDH, HRP-conjugated donkey anti-mouse, and HRP-conjugated donkey anti-rabbit. Antigen was detected by chemiluminescence (77).

### Immunofluorescence

Immunofluorescence was performed as previously described (68). Images were taken using Nikon i90 confocal microscope or Nikon Eclipse Ti2-E microscope equipped with a Yokogawa confocal scanner (CSU-W1). Primary antibody dilutions were as follows: 1:50 for phospho-Chk1 (Ser345); 1:500 for ATR; and 1:1000 for γH2AX (Ser139) and insulin. All secondary antibody dilutions were used at 1:1000.

### Nucleotide measurement

Nucleotides (ATP and NAD^+^) were quantified using HPLC analysis as previously described (78, 79). Briefly, following perchloric acid precipitation and centrifugation, supernatants were diluted with solvent A (0.1 M potassium phosphate and 4 mM tetrabutylammonium bisulfate [pH 6.0] 64:36 in water [v/v]), and nucleotide levels were quantified by HPLC using a SUPELCOSIL LC-18-T column (3 μm; 150 × 4.6-mm internal diameter). Total protein was determined on the precipitant using the Thermo Scientific Pierce BCA Protein Assay Kit. Nucleotide levels were normalized to total protein.

### Nitrite determination

Nitrite levels were determined using the Griess assay (80).

### Cellular bioenergetics

INS 832/13 cells (20,000 cells/well) and MEFs (10,000 cells/well) were plated in a Seahorse XF96 cell culture microplate, and extracellular flux was assessed using Seahorse XFe96 analyzer in DMEM containing 5.5 mM glucose, 2 mM pyruvate, and 1 mM glutamine. OCR and ECAR were measured in response to different mitochondrial respiratory inhibitors, and results were expressed as a change in percentage of the baseline for each cell type.

### Quantitative RT-PCR

Total RNA was isolated from INS 832/13 cells and rat islets using RNeasy Kit from QIAGEN. RNA samples were reverse transcribed to complementary DNA using oligo (dT) primer and reverse transcriptase (Thermo Fisher Scientific), following the manufacturer's instructions. Quantitative PCR was performed using the SsoFast EvaGreen supermix (Bio-Rad) and a Bio-Rad CFX96 Real-Time system. Primers specific for the following rat genes were purchased from Integrated DNA Technologies, and sequences were as follows: Atm forward 5′-CCA CGT CGT CTA TCG TTG GT-3′; Atm reverse 5′-AGT GAT ACC CAT CCC GTC CA-3′; Atr forward 5′-ACA ACA CTG CTG GCT TGA GA-3′; Atr reverse 5′-GTG CCT GGG CAG GAG TAT TT-3′; Trim28 forward 5′-GTG TGA GAC CTG TGT GGA GG-3′; Trim28 reverse 5′-CAA GGG GTT CAT GCT TGT GC-3′; Chek1 forward 5′-CTG GTT GAC TTC CGG CTT TC-3′; Chek1 reverse 5′-CCT TCT GGC TGC TCA CGA TA-3′; Gapdh forward 5′-GAC ATC AAG AAG GTG GTG AAG C-3′; and Gapdh reverse 5′-TCC AGG GTT TCT TAC TCC TTG G-3′. For absolute quantification, mRNA amounts were determined using standard curves generated from purified PCR products (81).

### siRNA transfection

siRNA transfection was performed using Lipofectamine 2000 according to the manufacturer's instructions (Invitrogen). Cells were reverse transfected with siRNA at a final concentration of 100 nM for 48 h, and medium was replaced prior to treatment. Knockdown efficiency was confirmed by quantifying target mRNA levels *via* quantitative RT-PCR.

### Statistical analysis

Statistical analysis was performed using *t* test and one-way or two-way ANOVA with a Tukey's multiple comparison post hoc test. Statistically significant differences (*p* < 0.05) are indicated with an asterisk.

### Data availability

All the data not contained in the article will be shared upon request to John A. Corbett, Medical College of Wisconsin, jcorbett@mcw.edu.

## Conflict of interest

The authors declare that they have no conflicts of interest with the contents of the article.
